# Practical Observations on Aural Surgery and the Nature and Treatment of Diseases of the Ear

**Published:** 1853

**Authors:** 


ART. XIV.—*Practical Observations on Aural Surgery and the Nature and Treatment of Diseases of the Ear.* With illustrations. By WILLIAM R. WILDE, Fellow of the Royal College of Surgeons in Ireland; Surgeon to St. Mark's Ophthalmic Hospital; Honorary Member of the Royal Medical Society of Stockholm, etc., etc. Philadelphia: Blanchard & Lea. 1853. Pp. 475, 8vo.

A good work on Diseases of the Ear has long been felt to be a desideratum in our medical literature. The pathology and treatment of such affections are so little understood by the profession that physicians have generally been content to surrender their cases of aural surgery to specialists, who have been too often little better than pretenders. No work has ever entered so extensively into the pathology and treatment of these diseases as the one of which we have given the title above, and in which the author has attempted to lay down just principles for an accurate diagnosis, and to found the treatment upon the well-established laws of modern pathology and reasonable therapeutics. It is a practical work, founded upon extensive experience, and therefore entirely trustworthy. It is precisely such a treatise as one wishes to possess, who has a case of disease of the ear before him, the character of which he does not fully comprehend.

Mr. Wilde is a surgeon of high reputation for learning and skill, and the hospital in which he has acquired his practical knowledge of diseases of the ear is an institution created by his own energy. His work is a seasonable contribution to our medical literature, and those enterprising publishers, Messrs. Blanchard & Lea, have done the profession of our country a favor by its republication.

